# Computational fluid dynamics analysis for predicting microaneurysm formation in parent arteries of unruptured cerebral aneurysms: implications for neck clipping safety

**DOI:** 10.3389/fneur.2025.1531703

**Published:** 2025-03-03

**Authors:** Kento Sasaki, Ichiro Nakahara, Kotaro Kihara, Shiho Tanaka, Riki Tanaka, Akiko Hasebe, Jun Tanabe, Kenichi Haraguchi, Yasuhiro Yamada, Fuminari Komatsu, Mai Okubo, Tomoka Katayama, Yoko Kato, Yuichi Hirose

**Affiliations:** ^1^Department of Neurosurgery, Fujita Health University Bantane Hospital, Nagoya, Aichi, Japan; ^2^Department of Neurosurgery, Fujita Health University, Toyoake, Aichi, Japan

**Keywords:** computational fluid dynamics analysis, microaneurysm, unruptured cerebral aneurysm, neck clipping, parent artery radiation sign

## Abstract

**Background:**

Aneurysmal subarachnoid hemorrhage caused by cerebral aneurysm rupture has a poor prognosis, with mortality exceeding 30% despite treatment advancements. Surgical neck clipping remains the standard for preventing rupture, but intraoperative rupture rates vary significantly (3–50%) and are influenced by vascular complexity and technical challenges. Thinning of the vascular wall near the aneurysm neck, particularly with microaneurysm formation, has emerged as a significant risk factor, yet these changes often go undetected in preoperative imaging.

**Objective:**

This study aimed to evaluate the utility of computational fluid dynamics (CFD) analysis for predicting microaneurysm formation in the parent artery adjacent to unruptured cerebral aneurysms, using the parent artery radiation sign (PARS) as a predictive marker.

**Methods:**

We conducted a single-center, retrospective observational study of 89 patients with unruptured middle cerebral artery (MCA) aneurysms treated with neck clipping from May 2020 to April 2022. Based on preoperative three-dimensional computed tomography angiography (3D-CTA), CFD analysis identified PARS through specific hemodynamic indicators. Intraoperative findings were analyzed and compared between PARS-positive and PARS-negative groups. The sensitivity and specificity of PARS for predicting microaneurysm formation were investigated.

**Results:**

Of the 87 aneurysms analyzed, 25 (28.7%) were PARS-positive, and 62 (71.3%) were PARS-negative. Microaneurysms were identified intraoperatively in nine cases, eight of which were in the PARS-positive group. The sensitivity and specificity of PARS for detecting microaneurysms were 89 and 78%, respectively. The positive likelihood ratio was 4.1, while the negative likelihood ratio was 0.142.

**Conclusion:**

CFD analysis using PARS offers a reliable method for predicting microaneurysm formation in the parent artery, potentially guiding surgical planning and reducing intraoperative rupture risk. While promising, these findings are limited by the retrospective, single-center design, highlighting the need for further research in larger, multicenter cohorts. Incorporating CFD analysis into preoperative assessment could significantly enhance the safety and outcomes of neck clipping procedures for unruptured cerebral aneurysms.

## 1 Introduction

Aneurysmal subarachnoid hemorrhage, resulting from the rupture of cerebral aneurysms, carries a poor prognosis exceeding 30% despite advancements in treatment options (1). Direct surgery to prevent rupture is recommended under specific conditions, with neck clipping as an established surgical technique. Intraoperative rupture, the most significant complication of neck clipping, occurs at rates ranging from 3 to 50% (2, 3). This variability depends on the complexity of the aneurysm’s vascular anatomy and the challenges of the surgical technique.

The risk of this complication is exceptionally high during the dissection phase of aneurysm exposure. Factors contributing to this include the technical difficulty of exposing the aneurysm, brain swelling, and the extent of manipulation during clipping (2, 3). The presence of a bleb in a cerebral aneurysm significantly increases the risk of intraoperative rupture during dissection (4). Furthermore, thinning of the vascular wall near the aneurysm neck, especially with microaneurysms, has been recognized to heighten intraoperative risk. These changes often elude detection in preoperative three-dimensional computed tomography angiography (3D-CTA) and become apparent intraoperatively, potentially confusing the surgeon and increasing procedural risk.

The number of reports related to computational fluid dynamics (CFD) analysis in the field of cerebral aneurysms has been increasing in recent years, and it is gaining more attention. There has been a substantial accumulation of research regarding CFD analysis specifically targeting cerebral aneurysms. These studies have primarily focused on aspects such as rupture risk, growth risk, hemodynamic changes related to treatment procedures, and post-treatment recurrence. Consensus has been gradually reached regarding parameters such as wall shear stress, wall pressure, and the vector of streamlines in aneurysms (5, 6). We have also conducted CFD analyses focusing on cerebral aneurysms themselves. Through our experience, we discovered abnormalities in CFD findings at sites of thinning of the parent artery wall and at locations where microaneurysms form. Based on these findings, we retrospectively analyzed the parent artery adjacent to cerebral aneurysms using preoperative 3D-CTA (7, 8) and defined this unique CFD finding as the “parent artery radiation sign” (PARS) (9). This study is significant because it focuses on morphological changes in the parent artery adjacent to the cerebral aneurysm.

However, previous studies with limited case numbers have not thoroughly examined the relationship between individual case factors and the significance of PARS in predicting microaneurysms preoperatively. Therefore, this study aims to investigate these aspects in detail, using a more extensive case series to evaluate the sensitivity and specificity of PARS for predicting microaneurysm formation in the parent artery near cerebral aneurysms.

## 2 Materials and methods

This single-center, retrospective observational study reviewed 89 cases of unruptured cerebral aneurysms treated with direct clipping between May 2020 and April 2022. Background factors compared included age, gender, aneurysm diameter, presence of multiple aneurysms, prior treated aneurysms, history of subarachnoid hemorrhage (SAH), family history of cerebral aneurysms, underlying conditions (hypertension, diabetes, dyslipidemia), statin use, and lifestyle factors (alcohol consumption and smoking habits). As in our previous report, aneurysm locations were limited to the bifurcation of the middle cerebral artery (MCA) to facilitate observation and ensure compatibility with computational fluid dynamics (CFD) analysis.

The CFD analysis method has been previously reported. In summary, blood flow impingement on cerebral aneurysm walls is associated with wall thinning (10). Streamlines visualizing blood flow pathways can identify impingement and turbulence sites, which may promote vessel wall thinning and aneurysm formation (11). PARS was defined by three CFD findings indicating blood flow impingement and elevated vessel wall pressure relative to surrounding areas: (1) streamline collisions with the vessel wall adjacent to the aneurysm; (2) radial dispersion of wall shear stress vectors at the same site; and (3) increased wall pressure. We compared intraoperative findings between the PARS-positive group (PARS group) and the PARS-negative group (control group).

Three-dimensional vascular images for CFD analysis were generated from CTA scans (TOSHIBA Aquilion One 320 columns) using the Ziostation2 image-processing workstation (Ziosoft, Tokyo, Japan) to extract blood vessels around the aneurysm. CFD images were then created using Hemoscope Project Manager 2015 (EBM/AMIN, Tokyo, Japan). Hemoscope has the advantage of enabling CFD analysis for target cases in a relatively short amount of time in clinical settings. There have been prior studies conducted by our research group as well as reports from other groups (12–14). Specifically, this software consolidates essential workflows for CFD analysis—including imaging, rendering, modeling, meshing, blood properties, boundary conditions, computation, visualization, and analysis—facilitating the full automation of CFD blood flow simulations. Prior to analysis, preparation involves extracting the region surrounding the target aneurysm from vascular images obtained via CTA. Upon launching the software and selecting either the steady flow or pulsatile flow mode, processes such as mesh generation, input of blood properties, setting of boundary conditions, selection of discretization schemes, and control of iterative computations are all automatically managed within the software. In this study, all CFD analyses were conducted in steady flow mode. Cases were excluded if they involved suspected dissecting aneurysms, incomplete CFD data, or non-circumferentially observed aneurysms. Four neurosurgeons familiar with CFD analysis and unruptured aneurysm surgery were randomly assigned to evaluate CFD findings and intraoperative outcomes related to wall shear stress vectors, with agreement rates calculated. Microaneurysms in the parent artery adjacent to the aneurysm were defined as microaneurysms located within 5 mm proximal or distal to the primary cerebral aneurysm. Discrepancies in PARS status or intraoperative microaneurysm findings were resolved through secondary CFD evaluation by a different assessor and an independent, blinded review of intraoperative findings. All procedures in this study adhered to the l9atest Declaration of Helsinki guidelines and were approved by the institutional ethics committee (No. HM24–061).

### 2.1 Data collection and statistical analysis

Laboratory data for all patients were obtained within 2 weeks before surgery. Additionally, statin medication history was extracted from the medication handbook, while patients’ self-reports were used to gather information on family history, medical history, alcohol consumption, and smoking habits. All statistical analyses were performed using R software (version 4.2.2).

## 3 Results

**Figure 1:**
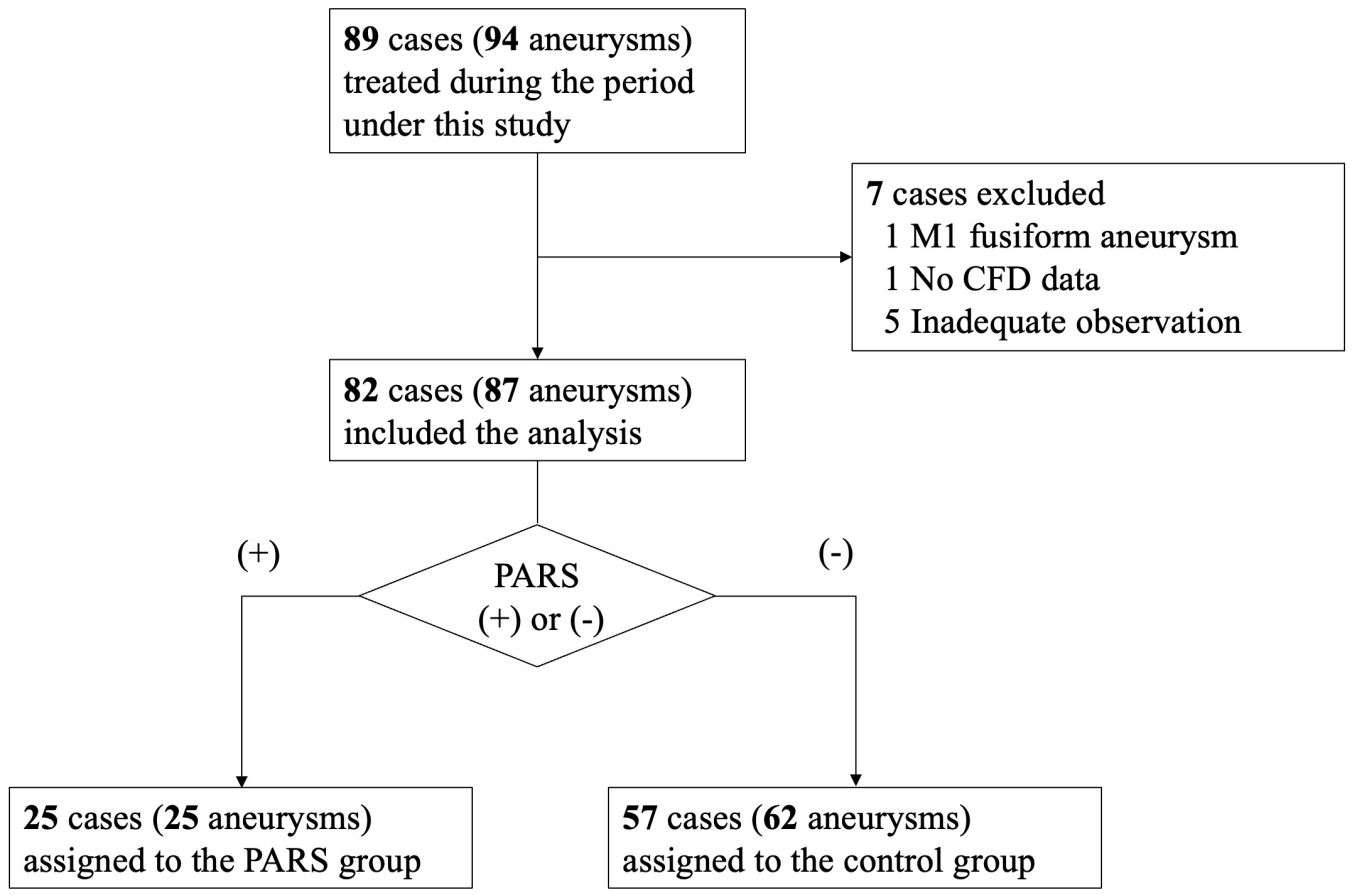
Patient selection flowchart.

During the study period, 89 patients with 94 unruptured middle cerebral artery (MCA) aneurysms underwent open clipping surgery. After applying the exclusion criteria, 82 patients with 87 aneurysms were included in the analysis, as depicted in the patient selection flowchart (Figure 1).

Background factors for the 82 patients are presented in Table 1.

**Table 1:** Background data of the 82 patients included in the study.

Variable	*N* = 82
Age (median[IQR])	71 (57.3–76.8)
Male (%)	16 (19.5)
Multiple aneurysm (%)	29 (35.4)
Treated history of other aneurysm (%)	18 (22.0)
SAH (%)	6 (7.3)
Family history of SAH (%)	11 (13.4)
HT (%)	52 (63.4)
DM (%)	13 (15.9)
DL (%)	28 (34.1)
History of statin agent taking (%)	24 (29.3)
Alcohol (%)	12 (14.6)
Smoking (%)	17 (20.7)

According to the CFD analysis results, 25 aneurysms (28.7%) were categorized as PARS-positive, while 62 aneurysms (71.3%) were classified as PARS-negative (control group).

Intraoperative findings identify microaneurysms on the parent artery adjacent to the aneurysm in nine of 87 MCA aneurysms, corresponding to an incidence rate of approximately 10% for microaneurysm formation on adjacent vessel walls. All cases with microaneurysm formation underwent additional clipping. Of the nine aneurysms with microaneurysm formation, eight were in the PARS group, while the remaining one was in the control group.

Table 2 summarizes the relationship between PARS positivity and the presence of microaneurysms on vessel walls adjacent to aneurysms. The sensitivity and specificity analysis of PARS positivity for microaneurysm detection yielded a sensitivity of 89% and a specificity of 78%. The positive likelihood ratio for PARS positivity in detecting microaneurysm formation on vessel walls was 4.1, while the negative likelihood ratio was 0.142.

**Table 2:** PARS in preoperative CFD analysis and intraoperative findings with/without microaneurysm of the adjacent mother vessel of the cerebral aneurysm.

	Microaneurysm (+)	Microaneurysm (−)	Total
PARS (+)	8	17	25
PARS (−)	1	61	62
Total	9	78	87

Table 3 presents a univariate logistic regression analysis comparing background factors with the presence of PARS in the nine aneurysms with microaneurysms. PARS positivity was statistically significant for microaneurysms, while no other factors showed statistical significance. Multivariate analysis could not be performed due to minimal intergroup differences for factors other than PARS and insufficient cases. For patients with multiple MCA aneurysms, the aneurysm size shown in the table corresponds to the larger aneurysm. However, separate analyses of the smaller aneurysm showed no statistically significant differences.

**Table 3:** Univariate analysis of the association between background factors and PARS With microaneurysm formation in the parent artery adjacent to cerebral aneurysms.

	*N* = 82		Microaneurysm *n* (%)	OR	95% CI		*p* value
					lower	upper	
Age^†^	<70	39	4 (10.3)	0.98	0.93	1.04	0.470
	≧70	43	5 (11.6)				
Sex	Male	16	2 (12.5)	1.20	0.17	5.66	0.828
	Female	66	7 (10.6)				
Multiple aneurysm	+	29	2 (6.9)	0.49	0.07	2.19	0.390
	−	53	7 (13.2)				
Treated history of other aneurysm	+	18	1 (5.6)	0.41	0.02	2.48	0.418
	−	64	8 (12.5)				
SAH	+	6	0 (0.0)	−	−	−	−
	−	82	9 (10.1)				
Family history of SAH	+	11	1 (9.1)	0.79	0.04	5.01	0.830
	−	71	8 (11.1)				
HT	+	52	3 (5.8)	0.24	0.05	1.01	0.061
	−	30	6 (20.0)				
DM	+	13	0 (0.0)	−	−	−	−
	−	69	9 (13.0)				
DL	+	28	1 (3.6)	0.21	0.01	1.25	0.155
	−	54	8 (14.8)				
History of statin agent taking	+	24	0 (0.0)	−	−	−	−
	−	58	9 (15.5)				
Alcohol	+	12	1 (8.3)	0.70	0.04	4.43	0.752
	−	70	8 (11.4)				
Smoking	+	17	1 (5.9)	0.45	0.02	2.70	0.461
	−	65	8 (12.3)				
Size of aneurysm^††^	<5 mm	33	2 (6.1)	1.25	0.87	1.77	0.209
	≧5 mm	49	7 (14.3)				
PARS	+	25	8 (32.0)	26.35^**†**^	4.40	507.14	0.003
	−	57	1 (1.8)				

^† The “Age” item was analyzed as a continuous variable.^

^†† The “Size of Aneurysm” item was analyzed as a continuous variable, with the larger aneurysm used for those with multiple aneurysms.^

### 3.1 Case presentations

Case 1: A 77-year-old woman with a right MCA aneurysm.

**Figure 2:**
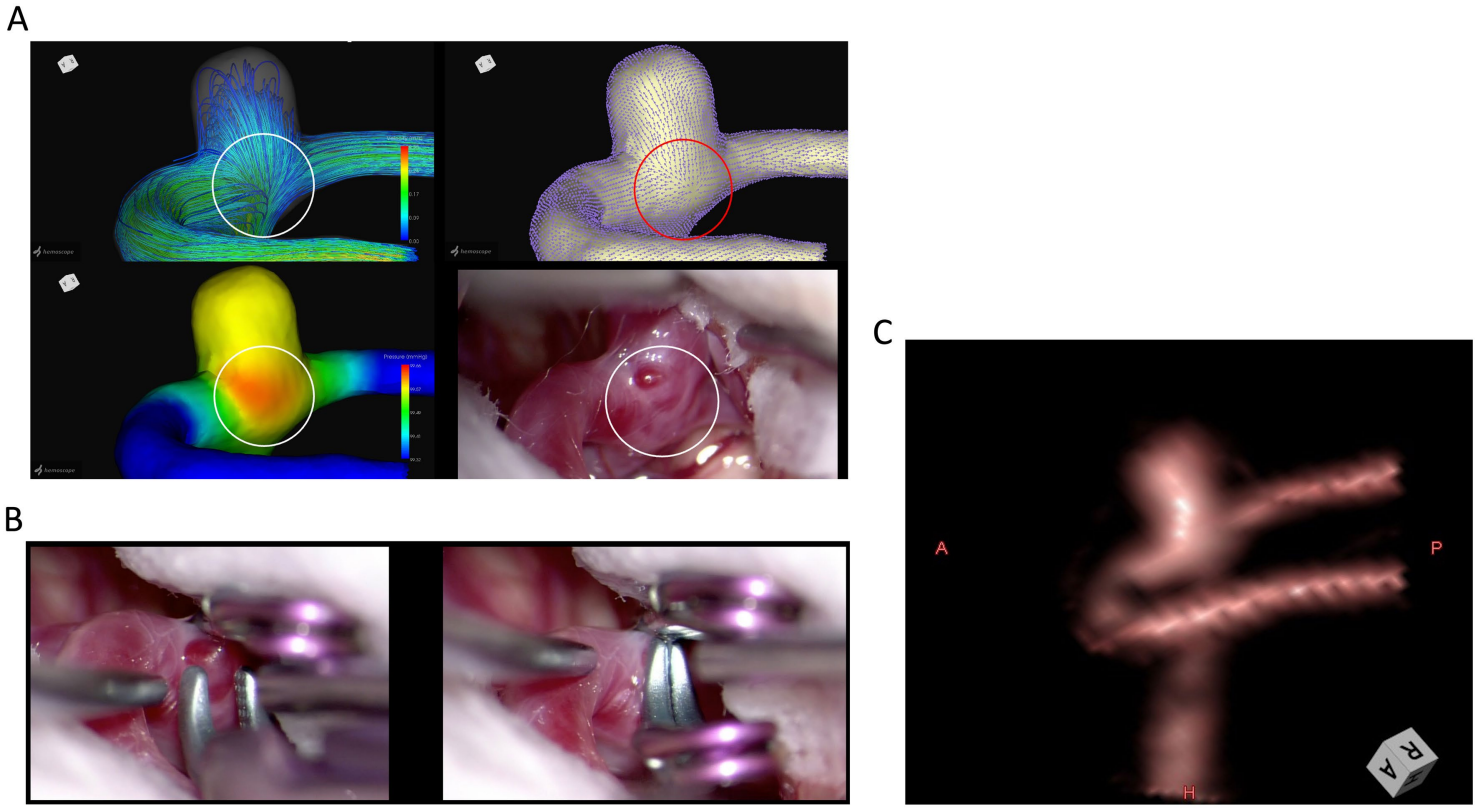
Case 1: A 77-year-old woman with a right MCA aneurysm. The CFD images and intraoperative view illustrate the radial dispersion of wall shear stress vectors and elevated wall pressure adjacent to the aneurysm within the encircled area **(A)**. The intraoperative view of additional clipping for a microaneurysm adjacent to the aneurysm **(B)**. Preoperative 3D-CTA image of the middle cerebral aneurysm at the angle of intraoperative findings **(C)**.

The CFD images and intraoperative view in Case 1 illustrate the radial spread of wall shear stress vectors and elevated wall pressure adjacent to the aneurysm within the encircled area (Figure 2). The intraoperative view reveals a microaneurysm (circled) adjacent to the aneurysm. The preoperative CTA showed no morphological changes at the same site.

Case 2: A 70-year-old woman with a right MCA aneurysm.

**Figure 3:**
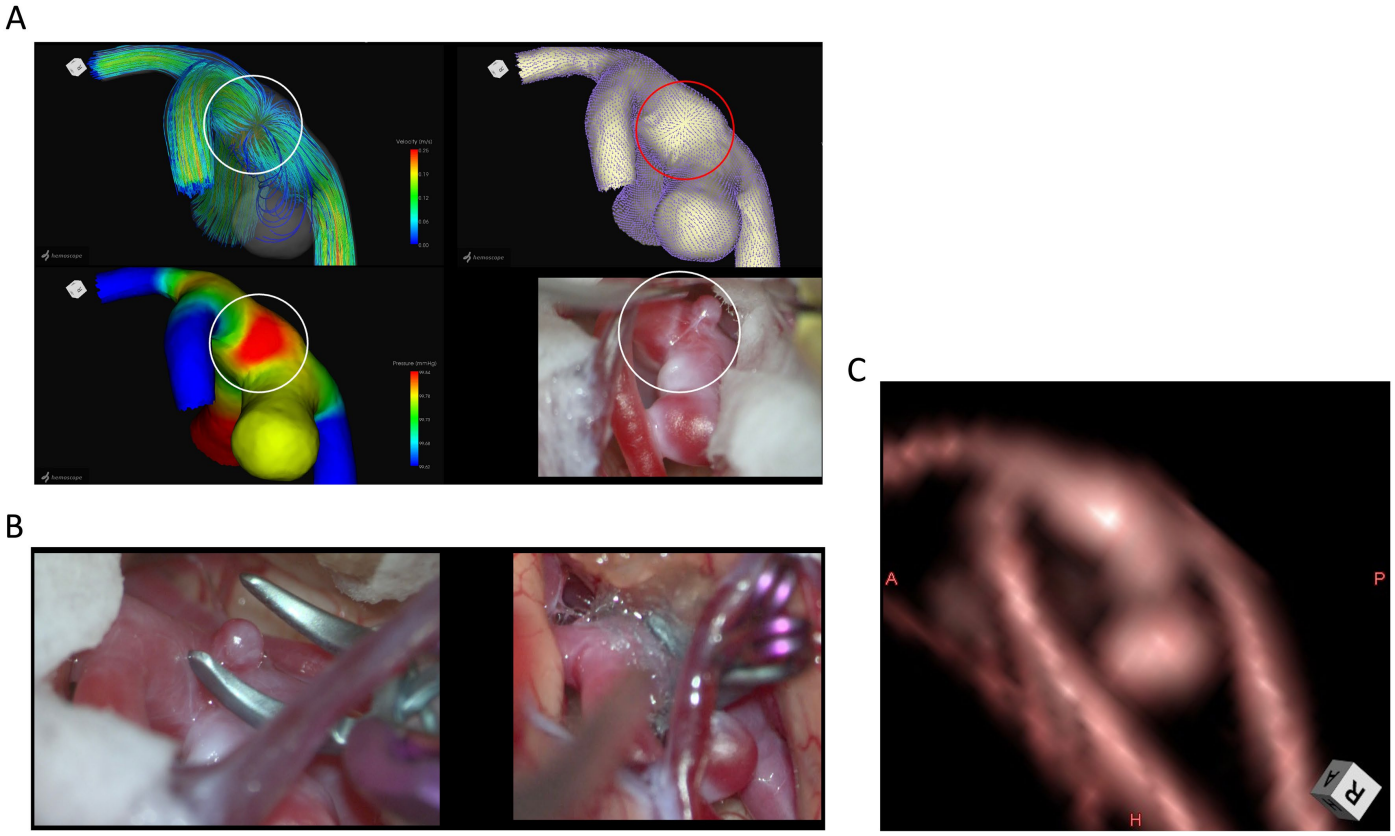
Case 2: A 70-year-old woman with a right MCA aneurysm. The CFD images and intraoperative view depict the radial dispersion of wall shear stress vectors and elevated wall pressure adjacent to the aneurysm within the encircled area **(A)**. The intraoperative view of additional clipping for a microaneurysm adjacent to the aneurysm **(B)**. Preoperative 3D-CTA image of the middle cerebral aneurysm at the angle of intraoperative findings **(C)**.

The CFD images and intraoperative view in Case 2 also illustrate the radial spread of wall shear stress vectors and the elevated wall pressure adjacent to the aneurysm within the encircled area (Figure 3). The intraoperative view similarly reveals a microaneurysm (circled) adjacent to the aneurysm. The preoperative CTA showed no morphological changes at the same site.

## 4 Discussion

Previous studies analyzing cerebral aneurysms using CFD have reported findings predicting wall irregularities of the aneurysm (15–17). In contrast, this study focuses on the vessel wall outside the aneurysm, demonstrating the potential of CFD analysis to predict wall thinning and microaneurysm formation in the parent artery near the aneurysm, which were not detected by preoperative vascular imaging. The radial dispersion of wall shear stress vectors indicates localized force application, particularly susceptible to microaneurysm formation (18). Thus, PARS may enhance sensitivity by highlighting areas where relatively strong forces are applied to the vessel wall. As defined in our previous research, PARS’s ability to predict unpredictable morphological changes in vessel walls with a sensitivity and specificity of 89 and 78%, respectively, is highly valuable for developing treatment strategies for unruptured cerebral aneurysms.

Assessing this method, alongside routine preoperative imaging, could be valuable in planning safe brain retraction and approaches to access the aneurysm, potentially improving surgical outcomes for unruptured cerebral aneurysms.

In recent years, endovascular treatments such as coil embolization, intrasaccular flow disruption, or flow diverter treatment have become more popular than surgical clipping for managing unruptured cerebral aneurysms (19–22)—however, coil embolization and intrasaccular flow disruption target only the aneurysm for occlusion. Flow diverter treatment, a device placed in the parent artery, may aid in repairing the parent artery near the aneurysm. Nonetheless, the use of this treatment for bifurcation aneurysms, such as the middle cerebral artery aneurysms in our study, remains debated (23, 24). Direct clipping may be preferable in cases like ours, where microaneurysms are present in the parent artery near the aneurysm neck (25). Thus, positive PARS findings from pre-treatment CFD analysis could aid in selecting appropriate treatment techniques.

### 4.1 Limitations

This was a single-center, retrospective observational study, and selection bias cannot be completely ruled out. Using CTA as the basis, detailed DSA might have detected subtle morphological changes in the vessel wall that CTA could not (26, 27). Based on deformed three-dimensional structures derived from preoperative CTA images, CFD analysis may overlook tiny aneurysms and perforating branches, potentially failing to capture true hemodynamics (27, 28). In this study, we used Hemoscope, a CFD analysis software package that is highly convenient and useful. However, it has the limitation of being unable to incorporate detailed parameters specific to individual cases, such as blood pressure and blood viscosity. To ensure analytical accuracy, this study focused on middle cerebral artery aneurysms, as evaluating blood flow and accurately depicting vessels at sites such as the anterior communicating artery or the internal carotid artery-posterior communicating artery bifurcation remains challenging. Additionally, CFD analysis is limited to angiographically visualized structures, which complicates its application to thrombotic or giant aneurysms (29, 30). Using only simple parameters may reduce accuracy, as different results could emerge if parameters such as oscillatory shear index, shear strain rate, pulsatility, and viscosity were included (13, 16, 31, 32).

## 5 Conclusion

This study demonstrates that computational fluid dynamics (CFD) analysis, mainly using the parent artery radiation sign (PARS), is valuable for predicting microaneurysm formation in the parent artery near unruptured cerebral aneurysms. With a sensitivity of 89% and specificity of 78%, PARS can guide preoperative planning for neck clipping, potentially reducing intraoperative rupture risk and improving surgical outcomes. Despite its promise, the study’s retrospective, single-center design presents limitations, necessitating further research with more extensive multicenter cohorts. Integrating CFD analysis into routine preoperative assessments may improve the safety and effectiveness of surgical interventions for cerebral aneurysms.

## Data Availability

The raw data supporting the conclusions of this article will be made available by the authors, without undue reservation.
